# Overcoming the pitfalls of automatic interpretation of whole genome sequencing data by online tools for the prediction of pyrazinamide resistance in *Mycobacterium tuberculosis*

**DOI:** 10.1371/journal.pone.0212798

**Published:** 2019-02-28

**Authors:** Tomotada Iwamoto, Yoshiro Murase, Shiomi Yoshida, Akio Aono, Makoto Kuroda, Tsuyoshi Sekizuka, Akifumi Yamashita, Kengo Kato, Takemasa Takii, Kentaro Arikawa, Seiya Kato, Satoshi Mitarai

**Affiliations:** 1 Department of Infectious Diseases, Kobe Institute of Health, Kobe City, Japan; 2 Bacteriology Division, Department of Mycobacterium Reference and Research, Research Institute of Tuberculosis, Japan Anti-Tuberculosis Association, Kiyose City, Tokyo, Japan; 3 Clinical Research Center, National Hospital Organization Kinki-chuo Chest Medical Center, Sakai City, Osaka, Japan; 4 Pathogen Genomics Center, National Institute of Infectious Diseases, Shinjuku-ku, Tokyo, Japan; 5 Molecular Epidemiology Division, Research Institute of Tuberculosis, Japan Anti-Tuberculosis Association, Kiyose City, Tokyo, Japan; 6 Research Institute of Tuberculosis, Japan Anti-Tuberculosis Association, Kiyose City, Tokyo, Japan; 7 Basic Mycobacteriosis, Nagasaki University Graduate School of Biomedical Sciences, Nagasaki City, Nagasaki, Japan; The University of Hong Kong, CHINA

## Abstract

**Objectives:**

Automated online software tools that analyse whole genome sequencing (WGS) data without the need for bioinformatics expertise can motivate the implementation of WGS-based molecular drug susceptibility testing (DST) in routine diagnostic settings for tuberculosis (TB). Pyrazinamide (PZA) is a key drug for current and future TB treatment regimens; however, it was reported that predictive power for PZA resistance by the available tools is low. Therefore, this low predictive power may make users hesitant to use the tools. This study aimed to elucidate why and to uncover the real performance of the tools when taking into account their variation calling lists (manual inspection), not just their automated reporting system (default setting) that was evaluated by previous studies.

**Methods:**

WGS data from 191 datasets comprising 108 PZA-resistant and 83 susceptible strains were used to evaluate the potential performance of the available online tools (TB Profiler, TGS-TB, PhyResSE, and CASTB) for predicting phenotypic PZA resistance.

**Results:**

When taking into consideration the variation calling lists, 73 variants in total (47 non-synonymous mutations and 26 indels) in *pncA* were detected by TGS-TB and PhyResSE, covering all mutations for the 108 PZA-resistant strains. The 73 variants were confirmed by Sanger sequencing. TB Profiler also detected all but three complete loss, two large deletion at the 3’-end, and one relatively large insertion of *pncA*. On the other hand, many of the 73 variants were lacking in the automated reporting systems except by TGS-TB; of these variants, CASTB detected only 20. By applying the ‘non-wild type sequence’ approach for predicting PZA resistance, accuracy of the results significantly improved compared with that of the automated results obtained by each tool.

**Conclusion:**

Users can obtain more accurate predictions for PZA resistance than previously reported by manually checking the results and applying the ‘non-wild type sequence’ approach.

## Introduction

*Mycobacterium tuberculosis* (MTB) is a slow-growing bacillus and thus requires several weeks to months for phenotypic drug-susceptibility testing (DST). The essential role and ubiquitous use of pyrazinamide (PZA) in tuberculosis (TB) therapy underlines the importance of DST against PZA [1,2]. However, the test for PZA is more complicated and less reliable than those for other anti-TB drugs due to the difficulty of growing TB under the acidic (pH < 6.0) conditions required for the assay [3–5]. As the current phenotypic test is difficult and time consuming, clinical laboratories are eager for a more accurate and rapid assay to detect PZA resistance. Genotypic DST is an ideal approach for bypassing the difficulty, inaccuracy, and lengthy process of phenotypic DST, providing rapid results [6,7]. However, genotypic DST for PZA is extremely challenging because the *pncA* mutations that induce loss of pyrazinamidase (PZase) activity (the major mechanism leading to PZA resistance) are highly diverse and scattered throughout the *pncA* gene [8,9].

Whole-genome sequencing (WGS) can theoretically be used to rule in resistance to all drugs, which would accelerate DST to design appropriate treatment regimens. To assist the interpretation of complex WGS data, several automated online software tools are available for TB DST, including CASTB [10], PhyResSE [11], TB Profiler [12], Mykrobe predictor [13], and TGS-TB [14]. These tools are expected to overcome the barrier of implementing WGS methodologies in routine diagnostic settings, which usually lack bioinformatics expertise. However, it has been reported that the sensitivity and specificity for predicting resistance varies significantly between the different tools and antibiotics tested [15]. Moreover, previous studies that evaluated the predictive accuracy of these automated tools for drug resistance revealed that the accuracy for predicting PZA resistance is low [12,15–17]. This may make users hesitant to use the tools.

Herein, we wanted to emphasise that a lack of drug resistance-associated variants in the automatically generated plain text report by the online tools does not necessarily mean the tools did not detect mutations in the region of interest, which are possibly causing resistance. Indeed, TB Profiler, TGS-TB, and PhyResSE provide variant calling lists in addition to the plain text report of drug resistance prediction no matter if they are included or not in the pre-defined mutation catalogue for automated reporting systems. Therefore, real performance evaluation of the tools should be performed by two different aspects, one at the level of the automated reporting system and the other at the full potential of its performance for the detection of mutations in a region of interest; all previous reports that evaluated online tool performance analysed the former aspect but did not take into account the latter [15–17].

To clarify the reasons for the reported low accuracy of PZA resistance prediction and to reveal the real performance of the tools, we evaluated the tools using WGS data from 191 datasets on resistant and susceptible strains at the level of automated reporting and the full potential of the tools.

## Materials and methods

### Specimens

A total of 191 MTB isolates collected from two different isolate stocks were used in this study. To avoid any unambiguous results by heteroresistance due to the existence of a mixed population [18], single colony isolation from the stock culture was performed. Sample set 1 included 112 multidrug-resistant MTB (MDR-TB) isolates from 112 patients, collected through the RYOKEN TB research consortium during 2012–2013 as part of a national drug resistance survey. Sample set 2 included 79 isolates obtained from Kinki-chuo Chest Medical Center located in the western part of Japan between 2001 and 2015; of these, 53 strains were MDR-TB. The isolates were obtained from 77 single patients including two cases where non-MDR-TB developed into MDR-TB in a single patient.

### Ethics statement

All of the clinical isolates in this study were fully anonymized before we accessed them. Clinical isolates of *M*. *tuberculosis* in set 2 were used with approval of the ethical committee of Kinki-chuo Chest Medical Center (reference 2015/493). Clinical isolates in set 1 were obtained through the survey followed Japanese national guidelines for epidemiological research and ethical approval was not required for this laboratory study based on *M*. *tuberculosis* isolates.

### Phenotypic DST of PZA

For all 191 strains, PZA susceptibility was measured using MGIT AST PZA (Becton Dickinson, Franklin Lakes, NJ) according to manufacturer’s instructions. The critical concentration used in this study was 100 μg/mL. Extended analysis using the PZase assay by Wayne’s method [19] was performed for the 16 strains that showed discrepant results between MGIT AST PZA and mutations in *pncA* or *rpsA* to determine PZA susceptibility. This analysis was also performed for all strains in sample set 1 (n = 112) to supplement MGIT AST PZA testing. Minimum inhibitory concentration (MIC) measurement for PZA was performed for 7 of the 16 strains whose PZA susceptibility could not be determined even after the PZase assay. MIC was determined [18] from PZA concentrations of 12.5, 25, 50, 100, 200, 300, 400, and 800 μg/mL in MGIT culture media, using the MGIT EpiCenter system. The MIC for *M*. *tuberculosis* H37Rv (control for PZA susceptibility) and *M*. *bovis* BCG Tokyo (control for PZA resistance) was found to be 50 μg/mL and > 800 μg/mL, respectively. We considered MIC values ≤ 100 as PZA susceptible.

### WGS data analysis

Genomic DNA of each isolate was prepared from MTB grown on Ogawa medium using an Isoplant Kit (Wako Pure Chemical Industries Ltd, Tokyo, Japan). A DNA-seq library was prepared with the QIAseq FX DNA library kit (Qiagen, Hilden, Germany) using 50 ng TB genomic DNA, followed by paired-end sequencing using Illumina MiSeq (Illumina, San Diego, CA) and the MiSeq Reagent Kit v3 (600 cycles) according to manufacturer’s instructions. The read data were then analysed using TB Profiler (online version, September 2018) [12], TGS-TB v2 [14], PhyResSE v1.0 [11], and CASTB v1.5 [10]. In addition to the text report obtained through automated interpretation (default result), we manually inspected the tool variation calling lists for *pncA*, *rpsA*, *panD*, and their promoter regions (manual result); this was not performed with CASTB as it does not provide a variation calling list. The synonymous *pncA* Gln122Gln mutation and the known phylogenetic informative mutations [12] *rpsA* Met432Thr (for LAM lineage) and *rpsA* Arg212Arg (for Beijing lineage) detected in this study were excluded from further analysis. Phenotypic PZA susceptibility testing (MGIT testing) complemented with the PZase assay and MIC measurement were used as the standard reference for evaluating the online tools’ prediction powers for PZA resistance.

### Sanger sequencing for *pncA*

To validate the mutation call by online tools, we performed sanger sequencing for all mutation variants of *pncA* detected in this study as previously described [20].

### Statistical analysis

All statistical analyses were performed with EZR [21], a graphical user interface for R.

### Read data deposit

Raw sequence reads of all 191 MTB strains subjected to WGS were deposited in the DDBJ Sequence Read Archive (DRA; https://www.ddbj.nig.ac.jp/dra/index.html) under the study accession number DRA006844 (S1 Table).

## Results

### Variation calling lists by online tools

Mutation variants detected in the 191 strains are summarised in Table 1.

**Table 1:** Mutation variants and detection by online tools.

Gene	Mutation	No. of variation	TB Profiler			TGS-TB			PhyRes			CASTB	
			Default	Manual	None	Default	Manual	None	Default	Manual	None	Default	None
*pncA*	SNV	47	35	12	0	42	5	0	33	14	0	10	37
	Indel*	26	0	20**	6	26	0	0	0	26	0	10	16
*rpsA*	SNV***	8	0	8	0	1	7	0	1	7	0	0	8
	Indel	1	0	1	0	0	1	0	0	1	0	0	1
*panD* promoter	SNV	1	0	1	0	0	1	0	0	1	0	0	1

None, not detected by either default or mannual inspection.

*Variation of the three complete and two 3' end deletions were discriminated based on their regions of deletion.

**Five deletions which showed discrepancy with TGS-TB and PhyRes were included (see S2 Table).

***Met432Thr is not included since it is a LAM defined SNP.

All mutations in *panA* detected through automated interpretation and manual inspection by three online tools (TB Profiler, TGS-TB, and PhyResSE) were validated by Sanger sequencing. Forty-seven single-nucleotide variants (SNVs) were detected by all three tools and were consistent with Sanger sequencing results. Regarding the indels, TGS-TB and PhyResSE called 26 variations that were consistent with Sanger sequencing, whereas TB Profiler showed different calls for 5 indel variants and no calls for 6 indels (three complete losses of *pncA*, two large deletions at the 3ʹ-end of *pncA*, and one relatively large insertion in *pncA*); details of these discrepancies are listed in S2 Table. Furthermore, eight SNVs and one indel in *rpsA* as well as one SNV in the *panD* promoter were detected by all three tools; S1 Fig shows an image of the detection of these variants by TGS-TB. In total, 83 variants (47 SNVs and 26 indels in *pncA*, 8 SNVs and 1 indel in *rpsA*, and 1 SNV in the *panD* promoter) were detected in this study and used for further analysis to evaluate the online tools’ prediction powers for PZA resistance. All strains with variants and phenotypic PZA susceptibility informationare listed in S1 Table.

### Determination of PZA susceptibility

Of the 191 strains analysed, 98 demonstrated resistance by the MGIT test with mutations in *pncA* and its promoter (60 in set 1 and 38 in set 2) and were judged PZA resistant (Table 2). The 77 strains that were deemed susceptible by MGIT and without mutations (40 in set 1 and 37 in set 2) were judged PZA susceptible. These classifications correspond with PZase analysis results (S1 Table).

**Table 2:** Association between PZA resistant conferring mutations and MGIT PZA susceptibility testing.

	MGIT (set 1, n = 112)		MGIT (set 2, n = 79)	
	R	S	R	S
Default mutations by TB-Profiler	35	5	21	4
Mutations other than default by TB Profiler	25	7	17	0
No mutations	0	40	0	37

PZA, pyrazinamide; R, resistant; S, sensitive

Meanwhile, 16 strains of 191 showed discrepant results between genotypic and phenotypic results (Table 2); nine strains were found negative by PZase analysis (the major mechanism leading to PZA resistance) with mutations in the *pncA* gene and were thus characterised as PZA resistant (Table 3).

**Table 3:** Extended analysis of the 16 *M*. *tuberculosis* strains for determining PZA susceptibility.

Strain ID	Source	Default mutations by TB Profiler	Mutations other than default by TB Profiler in candidate genes	MGIT PZA	PZase	MIC (μg/mL)	Conclusion
126	set 2	*pncA (Lys96Gln)*	none	S	neg.	N/A	R
135	set 2	*pncA (Asp63Gly)*	none	S	neg.	N/A	R
131	set 2	*pncA (Gly78Ser)*	none	S	neg.	N/A	R
020	set 1	*pncA (Leu182Ser)*	none	S	neg.	N/A	R
011	set 1	*pncA (T-11C promoter)*	none	S	neg.	N/A	R
018	set 1	*pncA (Lys96Asn)*	none	S	neg.	N/A	R
017	set 1	*pncA (Met175Ile)*	none	S	±	200	R
019	set 1	*pncA (Leu4Ser)*	*rpsA (Asp15Ala)*	S	neg.	N/A	R
129	set 2	*pncA (Gly97Asp)*	*rpsA (1834845*: *TCGCCGCCGCCG /TCGCCGCCG)*, *panD promoter (A-13G)*	S	neg.	N/A	R
052	set 1	none	*pncA (Ser67Stop)*	S	neg.	N/A	R
049	set 1	none	*pncA (Val44Ala)*	S	±	100	S
048	set 1	none	*rpsA (1834845*: *TCGCCGCCGCCG /TCGCCGCCG)*	S	pos.	50	S
047	set 1	none	*rpsA (Asp123Ala)*	S	pos.	50	S
042	set 1	none	*rpsA (Thr42Ala)*	S	pos.	100	S
043	set 1	none	*rpsA (Asp27Asn)*	S	pos.	100	S
041	set 1	none	*rpsA (Tyr109His)*, *pncA (Stop187Gly)*	S	±	50	S

R, resistant; S, sensitive; N/A., not applicable; neg., negative; pos., positive; ±, indeterminant

Of the remaining seven strains that were further analysed by MICs, six were deemed PZA susceptible and one was PZA resistant (Table 3). None of the 191 tested isolates possessed mutations in the *panD* gene. Overall, the tested isolates were phenotypically classified into 108 PZA-resistant and 83 PZA-susceptible isolates.

### Mutations in PZA resistance-conferring genes

All 108 PZA-resistant strains possessed mutations in the *pncA* gene which consists of 44 different non-synonymous mutations, 26 different indels, and one mutation in the promoter region (S3 Table). None of the 71 mutations were found in the 83 PZA-susceptible strains. Among these mutations, 61 genetic variants had single *pncA* mutations per strain, one variant showed double *pncA* mutations, nine variants exhibited double mutations in both *pncA* and *rpsA* genes, and one variant had triple mutations in *pncA*, *rpsA*, and the *panD* promoter. All 26 indels in the *pncA*-containing region detected in this study led to PZA resistance, whereas two non-synonymous mutations in *pncA* (Stop187Gly, Val44Ala) did not confer PZA resistance. None of the 108 PZA-resistant strains possessed mutations in either *rpsA* or *panD* alone, which are putative PZA resistance-conferring genes. Among the 83 PZA-susceptible isolates, one possessed a *pncA* Val44Ala mutation, four exhibited single mutations in *rpsA* (Thr42Ala, Asp27Asn, Asp123Ala, or deletion at 1824845), and one showed double mutations in *pncA* Stop187Gly and *rpsA* Tyr109His (Table 2).

*pncA* mutations in this study were found distributed across the entire gene (Table 4), as previously reported [7,22,23].

**Table 4:** Non-synonymous mutations in *pncA* and their presence in the published lists.

Mutation	PZA senositivity		*pncA* mutations in the published lists				
	no. of R	no. of S	Miotto [22]	Yadon [23]	Manson [24]	Miotto [25]	Farhat [26]
*pncA (Met1Leu)*	1	0	*no*	*yes*	*no*	*no*	*no*
*pncA (Ala3Glu)*	6	0	*yes*	*yes*	*no*	*yes*	*no*
*pncA (Leu4Ser)*	1	0	*no*	*yes*	*no*	*yes*	*yes*
*pncA (Gln10Arg)*	1	0	*yes*	*yes*	*no*	*yes*	*yes*
*pncA (Asp12Ala)*	3	0	*yes*	*no*	*no*	*yes*	*yes*
*pncA (Asp12Glu)*	1	0	*yes*	*yes*	*no*	*no*	*yes*
*pncA (Cys14Stop)*	1	0	*yes*	*yes*	*no*	*yes*	*no*
*pncA (Cys14Tyr)*	1	0	*no*	*yes*	*no*	*no*	*no*
*pncA (Tyr34Asp)*	8	0	*no*	*no*	*no*	*yes*	*yes*
*pncA (Tyr34Stop)*	1	0	*yes*	*no*	*yes*	*yes*	*no*
*pncA (His51Gln)*	1	0	*yes*	*no*	*yes*	*yes*	*no*
*pncA (Pro54Leu)*	3	0	*yes*	*yes*	*no*	*yes*	*yes*
*pncA (His57Gln)*	1	0	*yes*	*yes*	*no*	*no*	*no*
*pncA (Leu159Pro)*	1	0	*no*	*yes*	*no*	*no*	*no*
*pncA (Asp63Gly)*	1	0	*yes*	*yes*	*no*	*yes*	*yes*
*pncA (Ser67Stop)*	1	0	*no*	*yes*	*no*	*no*	*yes*
*pncA (Trp68Arg)*	1	0	*yes*	*yes*	*no*	*yes*	*yes*
*pncA (Thr76Pro)*	1	0	*yes*	*no*	*no*	*yes*	*yes*
*pncA (Gly78Ser)*	3	0	*no*	*no*	*no*	*no*	*no*
*pncA (Lys96Asn)*	2	0	*yes*	*yes*	*no*	*yes*	*no*
*pncA (Lys96Gln)*	1	0	*yes*	*yes*	*no*	*no*	*no*
*pncA (Gly97Asp)*	3	0	*yes*	*yes*	*no*	*yes*	*yes*
*pncA (Thr100Pro)*	1	0	*yes*	*no*	*no*	*no*	*yes*
*pncA (Tyr103Ser)*	1	0	*no*	*no*	*no*	*no*	*no*
*pncA (Tyr103Stop)*	1	0	*yes*	*yes*	*yes*	*yes*	*yes*
*pncA (Ser104Ile)*	2	0	*yes*	*no*	*no*	*no*	*no*
*pncA (Leu116Arg)*	1	0	*yes*	*yes*	*no*	*yes*	*no*
*pncA (Trp119Cys)*	1	0	*yes*	*no*	*no*	*no*	*no*
*pncA (Val125Gly)*	1	0	*yes*	*no*	*no*	*no*	*no*
*pncA (Val128Gly)*	1	0	*yes*	*no*	*no*	*yes*	*yes*
*pncA (Gly132Ala)*	1	0	*yes*	*yes*	*no*	*yes*	*no*
*pncA (Asp136Gly)*	1	0	*no*	*no*	*no*	*no*	*yes*
*pncA (Cys138Arg)*	1	0	*yes*	*yes*	*no*	*no*	*no*
*pncA (Val139Ala)*	2	0	*no*	*yes*	*no*	*yes*	*yes*
*pncA (Val139Met)*	1	0	*no*	*yes*	*no*	*no*	*yes*
*pncA (Gln141Pro)*	3	0	*yes*	*no*	*no*	*yes*	*yes*
*pncA (Ala146Thr)*	1	0	*no*	*yes*	*no*	*no*	*no*
*pncA (Val155Met)*	1	0	*yes*	*yes*	*no*	*no*	*no*
*pncA (Val163Gly)*	1	0	*no*	*no*	*no*	*no*	*no*
*pncA (Ser164Pro)*	1	0	*yes*	*yes*	*no*	*no*	*no*
*pncA (Leu172Pro)*	1	0	*yes*	*yes*	*no*	*yes*	*yes*
*pncA (Met175Ile)*	2	0	*yes*	*yes*	*no*	*yes*	*no*
*pncA (Val180Phe)*	1	0	*yes*	*yes*	*no*	*yes*	*yes*
*pncA (Leu182Ser)*	2	0	*yes*	*yes*	*no*	*no*	*yes*
*pncA (Stop187Gly)*	0	1	*no*	*no*	*no*	*no*	*no*
*pncA (Val44Ala)*	0	1	*no*	*no*	*no*	*no*	*no*

R, resistant; S, sensitive

All 45 non-synonymous mutations in *pncA*—except for three mutations (Gly78Ser, Thr103Ser, Val163Gly)—detected in this study from 108 PZA-resistant strains were included in either of five comprehensively validated mutation lists as high confidence mutations [22–26] (Table 4). Although these three mutations were not on the list, the strains carrying these mutations showed negative results by PZase analysis and resistance via the MGIT test (S1 Table). Two strains harbouring the Stop187Gly and Val44Ala mutations in *pncA* were determined as PZA-susceptible by the MGIT test and neither mutation was listed in the five published lists (Table 4). The MIC values for these strains were not greater than 100 μg/mL, whereas *M*. *tuberculosis* H37Rv and *M*. *bovis* BCG Tokyo showed MICs of 50 μg/mL and > 800 μg/mL, respectively.

### Sensitivity and specificity of PZA resistance prediction by online tools

Using WGS data from 191 strains, we analysed the sensitivity and specificity of PZA resistance predictions by different tools available online (TB profiler, TGS-TB, PhyResSE, and CASTB). To evaluate tool performance at the automated reporting system level and at their full potential for mutation detection, we categorised the mutations detected by TB profiler, TGS-TB, and PhyResSE as either default or manual (S3 Table and Table 1); default indicates the automated interpretation report and manual indicates all mutations detected by the tools other than default.

Remarkable differences between the tools were found in their detection of indels. TGS-TB returned all 26 indel variants in the *pncA* gene through default settings, whereas the other two tools did not (Table 1). The default detection rate of SNVs in *pncA* varied among the tools; TGS-TB exhibited the best detection rate (42/47), followed by TB Profiler (35/47), PhyResSE (33/47), and CASTB which detected only 10 SNVs and 10 indels (Table 1). As a result, the default sensitivity of the tools was low (60.1%, 49.1%, and 31.5% for TB Profiler, PhyResSE, and CASTB, respectively), except for TGS-TB (97.2%; Table 5) which is consistent with previous studies [12,15–17].

**Table 5:** Sensitivity and specificity of the online tools in predicting PZA resistance.

			PZA susceptibility		Sensitivity (95% CI)	Specificity (95% CI)	PPV (95% CI)	NPV (95% CI)
			R (n = 108)	S (n = 83)				
Defalt setting	TGS-TB	R	105	1	0.972 (0.921–0.991)	0.988 (0.935–0.998)	0.991 (0.949–1.00)	0.965 (0.900–0.993)
		S	3	82				
	TB Profiler	R	65	0	0.601 (0.510–0.694)	1 (0.935–1.000)	1.000 (0.918–1.000)	0.659 (0.569–0.741)
		S	43	83				
	PhyResSE	R	53	1	0.491 (0.396–0.585)	0.988 (0.935–0.998)	0.981 (0.901–1.000)	0.599 (0.511–0.681)
		S	55	82				
	CASTB	R	34	1	0.315 (0.227–0.402)	0.988 (0.935–0.998)	0.971 (0.851–0.999)	0.526 (0.444–0.606)
		S	74	82				
Manual setting	TGS-TB	R	108	6	1 (0.950–1.000)	0.928 (0.872–0.983)	0.947 (0.889–0.980)	1.000 (0.931–1.000)
		S	0	77				
	TB Profiler	R	102	6	0.944 (0.883–0.979)	0.928 (0.849–0.973)	0.944 (0.883–0.979)	0.928 (0.849–0.973)
		S	6	77				
	PhyResSE	R	108	6	1 (0.950–1.000)	0.928 (0.872–0.983)	0.947 (0.889–0.980)	1.000 (0.931–1.000)
		S	0	77				

R, resistance; S, Sensitive; CI, confidence interval; PPV, positive predictive value; NPV, negative predictive value

All strains with indels or mutations detected by manual setting are classified as PZA resistance.

This drastically changed when we evaluated the detection rate with manual settings. PhyResSE detected all 26 indels while TB Profiler missed 6 indels (Table 1); of these 6 indels, two were large deletions at the 3ʹ region of *pncA*, three were complete losses of *pncA*, and one was a large fragment insertion at 2288788 (S3 Table). Five indels that were miscalled by TB Profiler (S2 Table) were treated as the call for PZA resistance prediction by TB Profiler. All mutations in *rpsA* and the *panD* promoter in our sample set were detected by the three tools through searches beyond the default report (Table 1). When we deemed the strains with these indels and non-synonymous mutations as PZA resistant (interpret ‘non-wild type sequence’ as PZA resistant), the three online tools demonstrated remarkably high sensitivity and specificity in predicting PZA resistance (Table 5). Specificity slightly decreased when manual results were compared with those of default (Table 5). This is due to the inclusion of mutations in *rpsA* whose association with PZA resistance is debatable [27,28].

## Discussion

Our analysis of online tool performance in detecting PZA resistance demonstrated that the tools can predict resistance with more sensitivity than previously reported when all detected variants (non-synonymous mutations and indels) are taken into account (Table 5). In fact, all or most mutations in the *pncA* gene of 108 PZA-resistant strains were detected by TB Profiler, TGS-TB, and PhyResSE but many variants were missing in the automated interpretation for plain text report. As a result, the accuracy of PZA resistance prediction by online tools differed substantially between the default report and manual inspection (Table 5). Thus, the fundamental cause for the reported low sensitivity of PZA resistance prediction is the tool interpretation pipeline, which is built upon pre-defined mutation catalogues and not due to the inability of software algorithms in detecting genetic variants.

The main aim of the online tools is to make complex WGS data analysis accessible for routine use in clinical settings where there is usually a lack in bioinformatics expertise. The advantage of these tools is automated interpretation for drug resistance upon pre-defined mutation catalogues. However, this study emphasised that proper knowledge of drug resistance-conferring mutations with some background in bioinformatics is still needed to avoid misinterpretations, i.e., a lack of drug resistance-associated variants in the automated report does not necessarily mean that there are no mutations in the region of interest causing resistance. Users should be aware that three online tools (TB Profiler, TGS-TB, PhyResSE) used in this study provide variant calling lists in addition to the plain text report of drug resistance prediction no matter if they are included or not in the pre-defined mutation catalogue for automated reporting systems. The previously reported low accuracy of PZA resistance prediction can be improved by a manual inspection of the list (Table 5). Although the tools do not guide users for this adequately, the variation calling list provided by the tools is a straightforward approach.

A recent genome-wide association study (GWAS) indicated that PZA resistance prediction can be enhanced by the inclusion of insertions and deletions [29], which was also confirmed by our data. It is clear that the superiority of TGS-TB over the other tools regarding the default setting was mainly due to the inclusion of indels for reporting PZA resistance. Our data showed a higher ratio of strains with structural variants by indels in PZA resistance (29.6%) compared with that of the previously reported values (16%, 19.6%, and 22.7%) for global samples [7], the Chinese province Zhejian [20], and the Chinese city Hanzhou [30], respectively. This higher ratio was observed for both sample set 1 (28.3%) and set 2 (30.0%) in our study, thereby reflecting the situation in Japan. These data imply that the impact of structural variants on PZA resistance is more significant in Japan where the ancient Beijing genotype is predominant compared with that of other regions [31,32]. Further studies are needed to clarify this distribution.

In this study, we interpreted strains with any non-synonymous mutations or indels in *pncA* as genotypic PZA-resistant strains; this ‘non-wild type sequence’ approach was implemented in the Genoscholar PZA-TB II line probe assay (PZA-LPA2; NIPRO Corporation, Osaka, Japan) [28]. One concern with this approach is the overestimation of resistant strains caused by non-resistance-associated mutations, including synonymous and lineage-specific mutations. Unlike the line probe assay, WGS-based DST can exempt them from mutations conferring PZA resistance, but with a lack of reliable lists as a drawback. Yadon et al. [23] identified many non-synonymous amino acid substitutions that do not confer PZA resistance by using a comprehensive library of *pncA* mutations in *M*. *tuberculosis* created by random PCR mutagenesis. Although these mutations were created *in vitro*, such a list would be helpful in identifying exemptions from the ‘non-wild type sequence’ approach. To evaluate the impact of non-resistance-associated mutations on this approach in clinical settings, we examined recent studies performed in clinical settings under well-controlled conditions. The degree of adverse effects by non-resistance-associated mutations varied but was low, i.e., one mutation out of 400 PZA-susceptible isolates (1/400) [3], 5/156 [20], 0/57 [28], and 2/83 in this study. This implies that the occurrence of such mutations inside a patient’s body is rare. Although the overestimation of resistant strains is unavoidable by the ‘non-wild type sequence’ approach, it can effectively screen PZA-susceptible strains and help reduce the need for culture-based DST, which is labour-intensive, requires facilities with biosafety measures, and is usually less reliable for PZA than other key drugs [33]. Inclusion of other PZA resistance-associated genes such as *panD* [34] may improve the accuracy of screening for PZA-susceptible strains, although it was not detected in this study. In our sample set, inclusion of mutations in *rpsA* as PZA resistance-conferring mutations decreased specificity but did not increase sensitivity of WGS-based tools for PZA resistance prediction (Table 5). It remains debatable if *rpsA* is associated with PZA resistance [27,28]. Moreover, to draw conclusions on its use for the prediction of PZA resistance, MIC measurement for a large number of strains with mutations in *rpsA* should be performed.

It is of note that detection of a complete region of interest deletion, such as for *pncA* in this study and *katG* for isoniazid resistance [35], is troublesome. TGS-TB and PhyResSE can detect complete deletions; however, we had to check the mapping status of PhyResSE results, which is not straightforward from the variation calling list and would likely be missed. TGS-TB was much more user friendly in this regard. TB profiler did not report the deletion as the tool has no function to visualise mapping status. To make the tools more user friendly, the developers should consider reporting the intactness of the region of interest as ‘intact and wild-type’ and including it in the automated reporting system, especially for *pncA* and possibly *katG* as well.

The study limitation is that we did not evaluate heteroresistant strains of mixed populations that appear in clinical settings [18]. How to deal with phenotype/genotype discrepant results, mutations conferring low levels of resistance, and heteroresistance are the practical questions that need to be addressed by WGS data analysis [36,37]. In conclusion, our findings demonstrated that users can obtain more accurate predictions for PZA resistance than previously reported by carefully checking the results manually and applying the ‘non-wild type sequence’ approach. It would be worthwhile for developers to include all *pncA* mutations without synonymous and lineage-specific mutations into their automated reporting system for PZA resistance prediction as already implemented by TGS-TB for indels. This makes online PZA resistance prediction tools more suitable for clinical setting where there is usually a lack in bioinformatics expertise.

## Supporting information

S1 Fig Capture image of mapping results by TGS-TB. (PPTX)

S1 Table Strains analyzed in this study and their profiles of PZA resistance. (XLSX)

S2 Table Variants in *pncA* those showed discrepant mutation calls among the online tools. (XLSX)

S3 Table Variation of the mutations detected in this study. (XLSX)
