# Supplementary material for: Overcoming the pitfalls of automatic interpretation of whole genome sequencing data by online tools for the prediction of pyrazinamide resistance in Mycobacterium tuberculosis
Source: PLoS One. 2019 Feb 28;14(2):e0212798. doi: 10.1371/journal.pone.0212798 (PMC6394917; doi:10.1371/journal.pone.0212798)
Supplement: S1 Fig — (PPTX) [file pone.0212798.s001.pptx]

## Slide 1
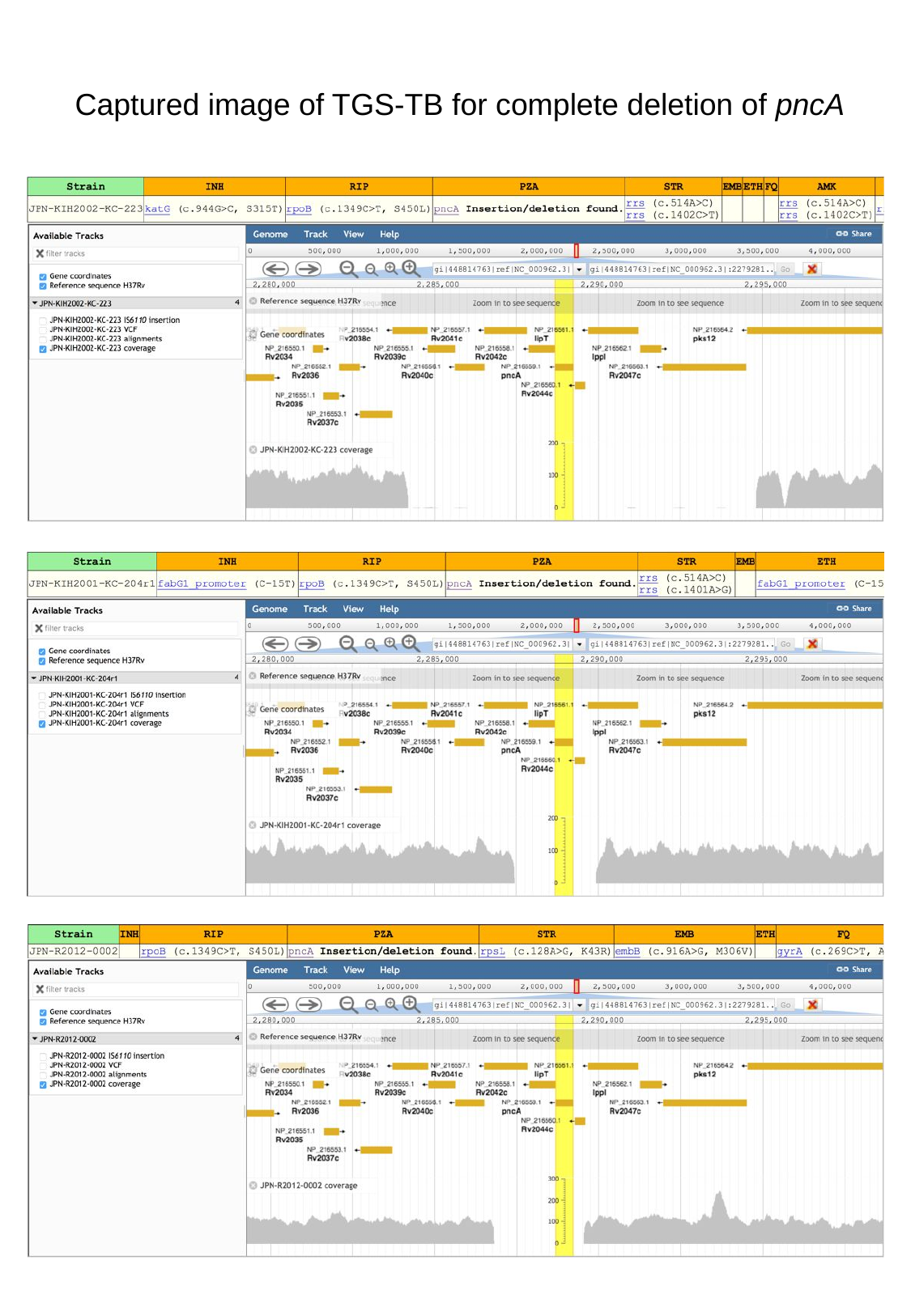

Captured image of TGS-TB for complete deletion of pncA

## Slide 2
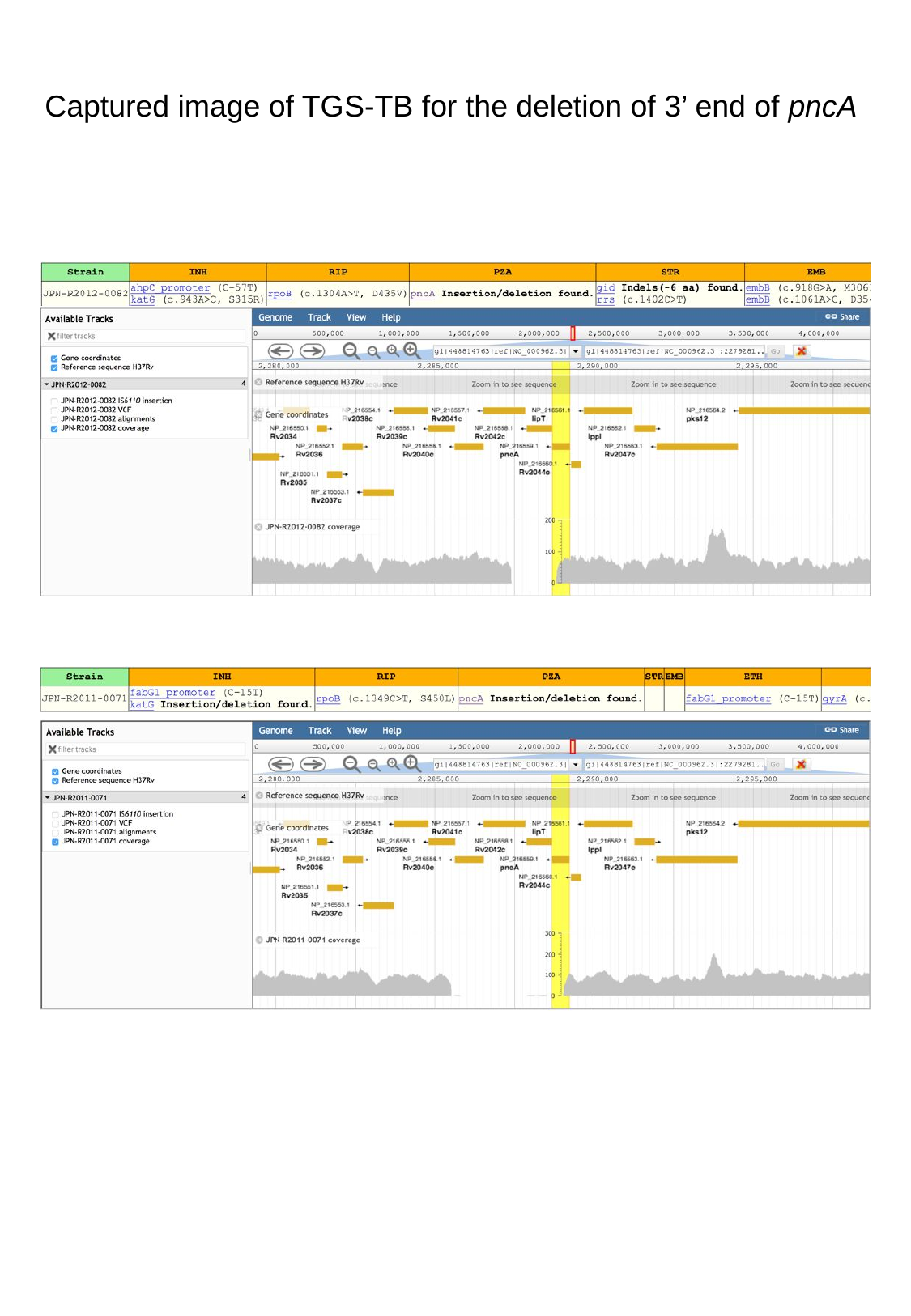

Captured image of TGS-TB for the deletion of 3’ end of pncA

## Slide 3
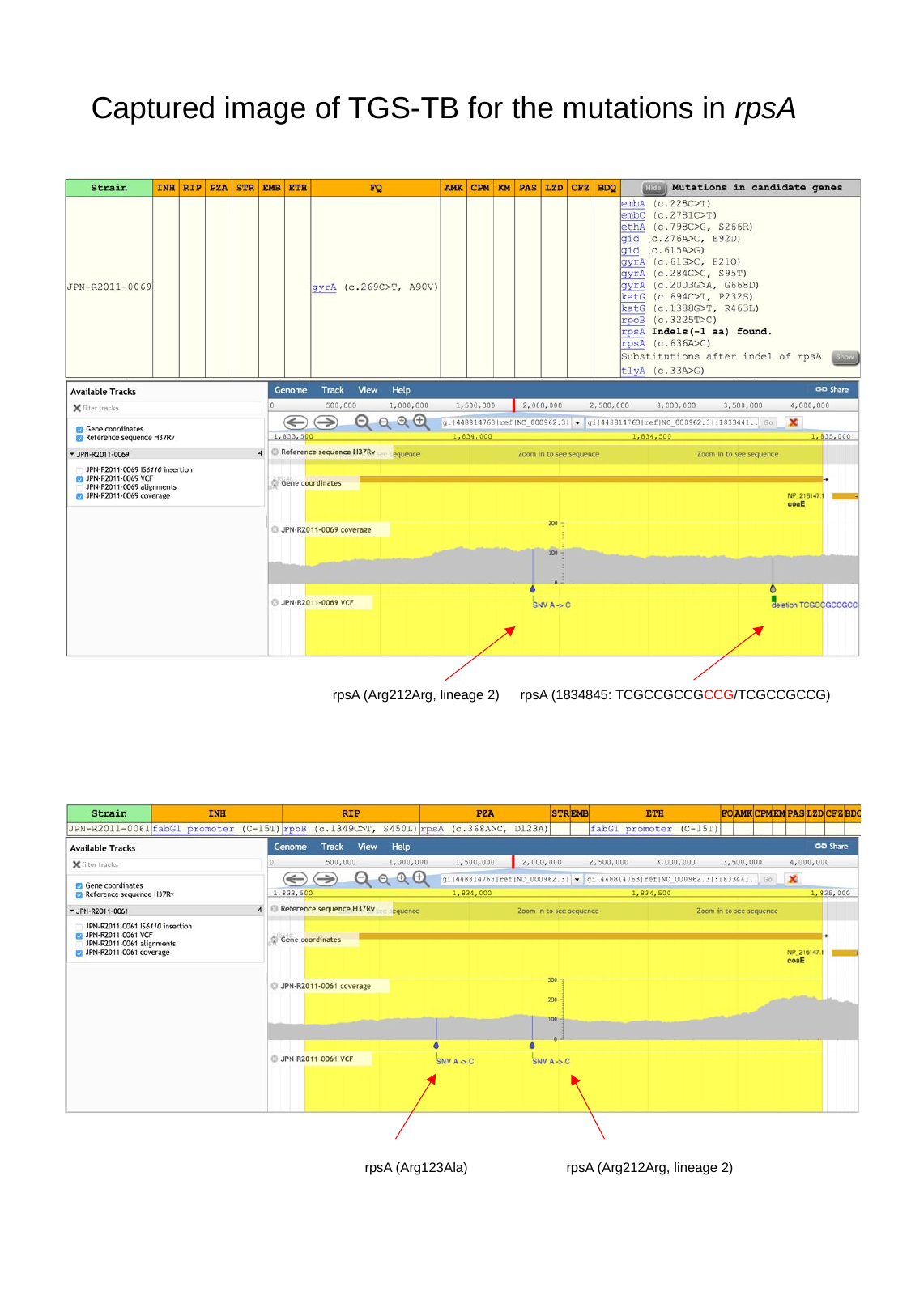

Captured image of TGS-TB for the mutations in rpsA
rpsA (Arg212Arg, lineage 2)
rpsA (1834845: TCGCCGCCGCCG/TCGCCGCCG)
rpsA (Arg123Ala)
rpsA (Arg212Arg, lineage 2)

## Slide 4
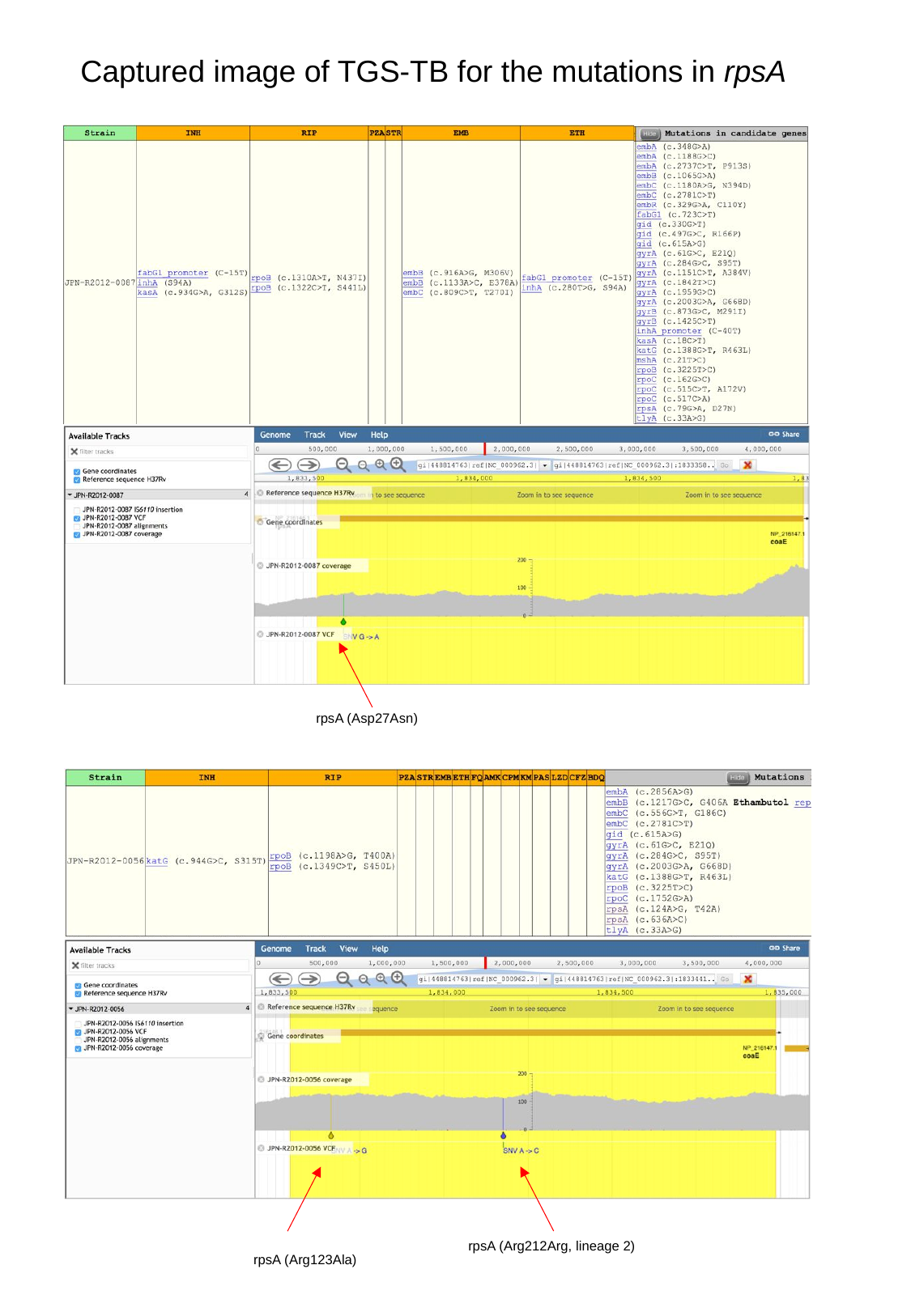

Captured image of TGS-TB for the mutations in rpsA
rpsA (Asp27Asn)
rpsA (Arg212Arg, lineage 2)
rpsA (Arg123Ala)

## Slide 5
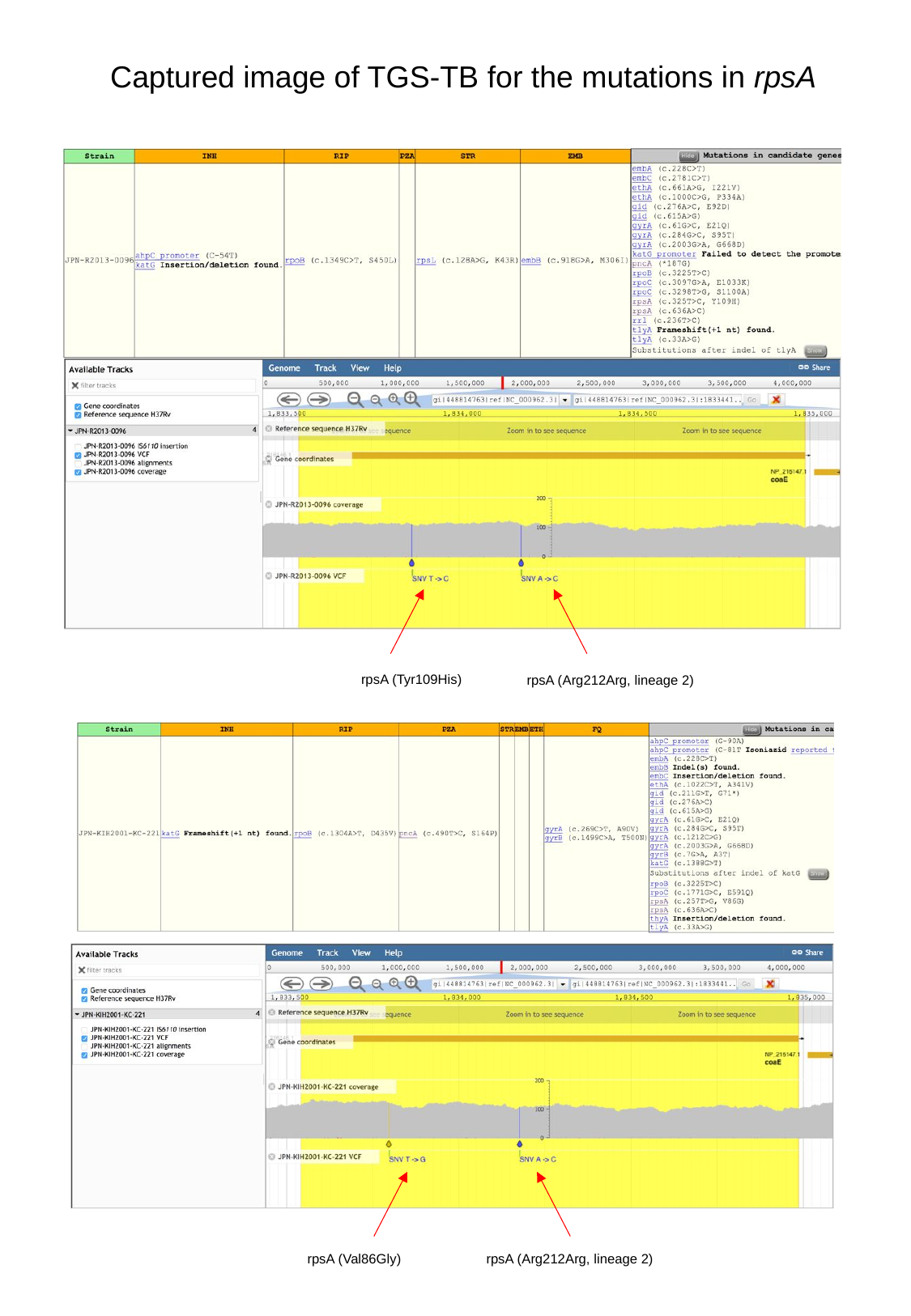

Captured image of TGS-TB for the mutations in rpsA
rpsA (Tyr109His)
rpsA (Arg212Arg, lineage 2)
rpsA (Val86Gly)
rpsA (Arg212Arg, lineage 2)

## Slide 6
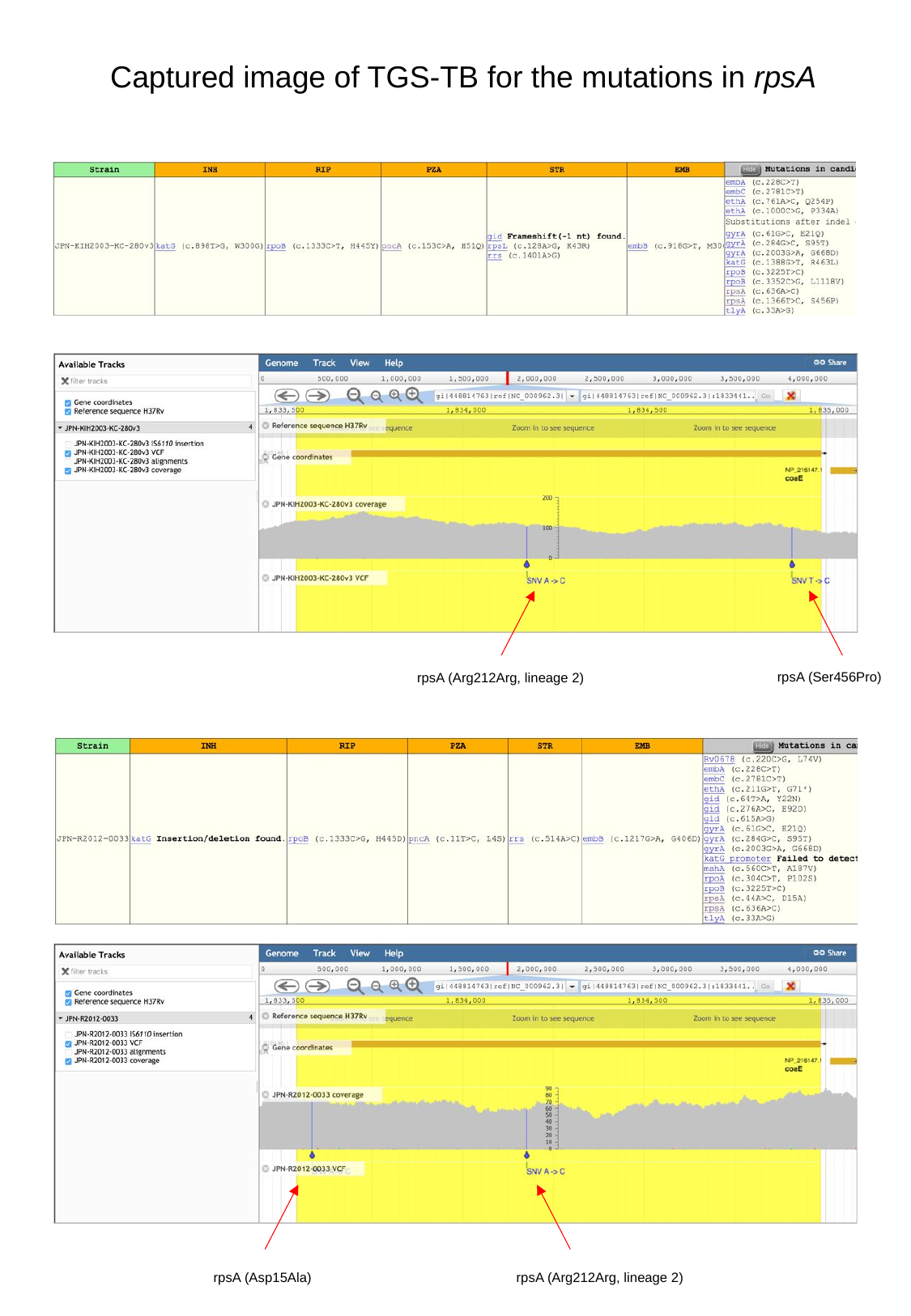

Captured image of TGS-TB for the mutations in rpsA
rpsA (Ser456Pro)
rpsA (Arg212Arg, lineage 2)
rpsA (Asp15Ala)
rpsA (Arg212Arg, lineage 2)

## Slide 7
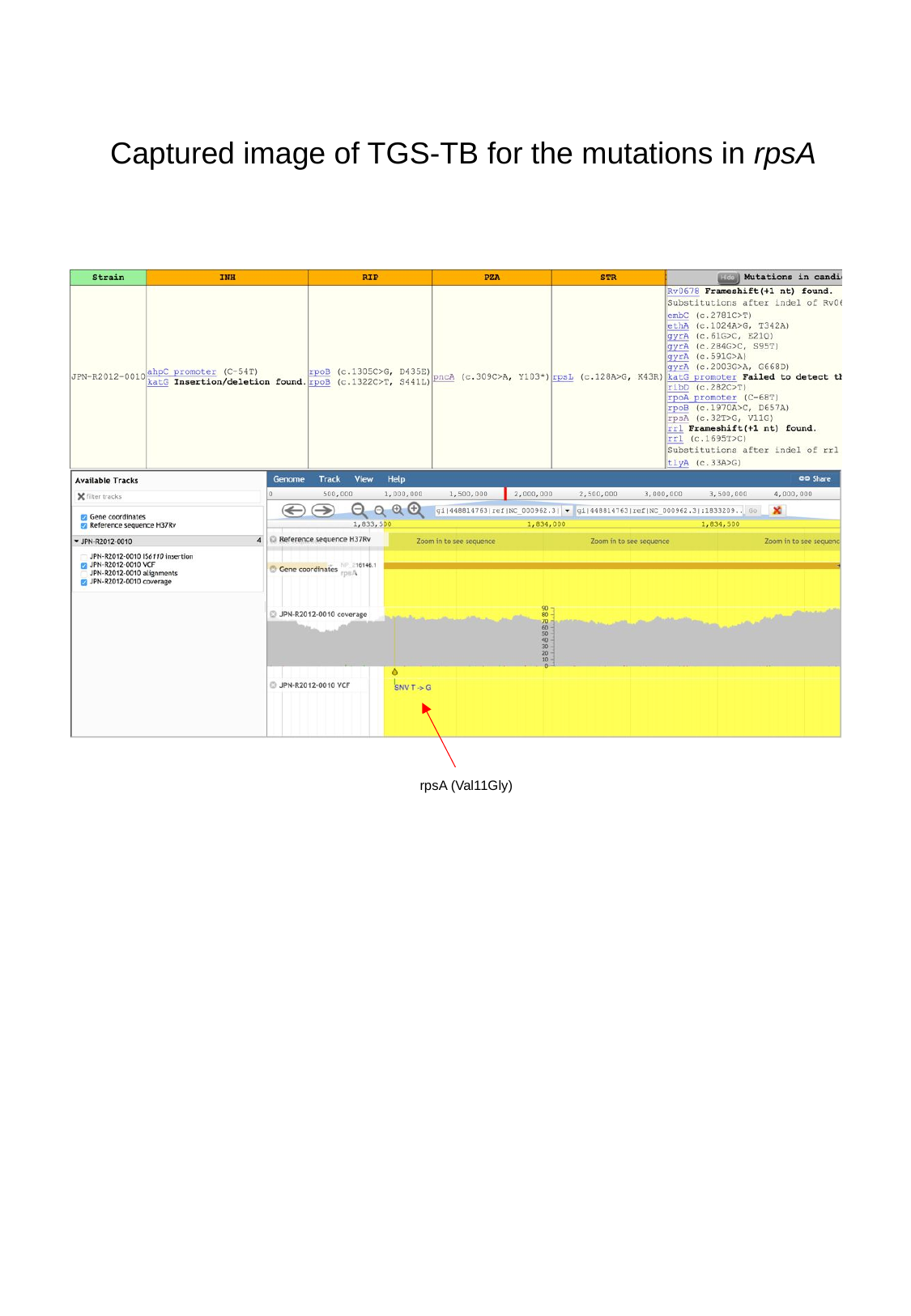

Captured image of TGS-TB for the mutations in rpsA
rpsA (Val11Gly)

## Slide 8
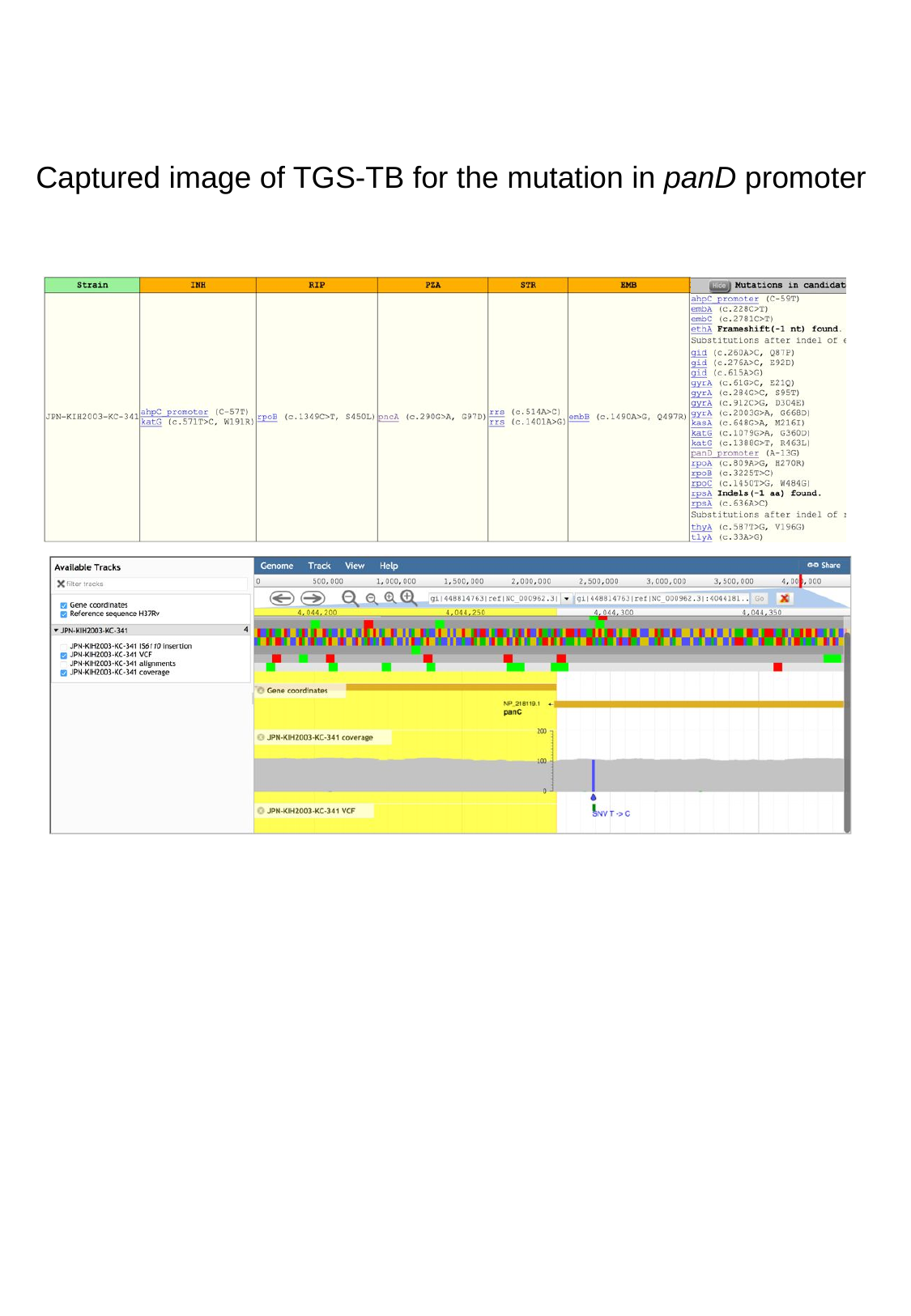

Captured image of TGS-TB for the mutation in panD promoter
